# Intussusception in an Infant Cured by Inflammation of the Bowel

**Published:** 1871-01

**Authors:** 


### INTUSSUSCEPTION IN AN INFANT CURED BY INFLATION OF THE BOWEL.

W. S., aged six months, admitted into Guy's Hospital March 28, 1870. The child appeared in perfect health until yesterday afternoon about four o'clock, when, while sucking a crust of bread, he suddenly screamed out, fainted, and became cold. The mother took him to a doctor, who gave him a powder, which made him very sick. He continued in great pain, and cried incessantly. At three o'clock this morning he passed a quantity of clotted blood per rectum, and this continued to run from him until he was admitted into the hospital, at 12 o'clock. The last fæcal evacuation took place at noon the previous day.

On admission, the child was seen to be well grown, but face pale, and had a generally collapsed appearance. On examining his abdomen, a lump was distinctly felt to the left and above the umbilicus, which hardened when pressed upon. On passing the finger up the rectum, a round projection could be felt about four inches up, with a circular orifice in the centre. The finger, when withdrawn, was covered with blood. The case being thus clearly one of intussusception, Dr. Wilks ordered inflation of the bowel by means of a bellows. Chloroform was given, and an enema tube passed into the rectum, the other end being attached to the bellows. The attempt to inflate was at first unsuccessful, owing to the large size of the rectum; but by increasing the width of the tube by wrapping a strip of lint round it, the colon was well inflated, and then the lump gradually went back until it quite disappeared. A drop of [tr.?] opium was ordered in a drachm of dill-water, and the breast to be given sparingly.

On the following day, March 29th, no lump could be felt. The child had been sick several times, and nothing had passed per rectum. To repeat the medicine.

March 30th, child very irritable; apparently much tenderness over abdomen, especially toward the right side. Occasionally sick. Passed a little blood, but no fæces.

31st, evidently better. Had a liquid evacuation with no blood, and sucks well.

April 1st, passed a natural motion, and altogether better.

2d, child apparently well, and taken out by the mother, who was somewhat discontented at the operation performed on him, as she never could be made to realize the severity of the case.

He remained well until the 10th, when he was brought to the hospital, having had fresh bleeding, and the lump could again be felt. Ths mother would not allow the child to be again taken in for the purpose of a renewal of the method which had been before so successful, but took him away for the purpose of procuring some physic for him; and no more was heard of the case.—*Lancet*, May 21, 1870.

